# Neonatal hyperglycaemia is associated with worse neurodevelopmental outcomes in extremely preterm infants

**DOI:** 10.1136/archdischild-2020-319926

**Published:** 2021-04-16

**Authors:** Itay Zamir, Elisabeth Stoltz Sjöström, Fredrik Ahlsson, Ingrid Hansen-Pupp, Fredrik Serenius, Magnus Domellöf

**Affiliations:** 1 Department of Clinical Sciences, Pediatrics, Umeå University, Umeå, Sweden; 2 Department of Food, Nutrition and Culinary Science, Umeå University, Umeå, Sweden; 3 Department of Women's and Children's Health, Pediatrics, Uppsala University, Uppsala, Sweden; 4 Department of Clinical Sciences Lund, Pediatrics, Lund University, Skåne University Hospital, Lund, Sweden

**Keywords:** endocrinology, neonatology, neurology

## Abstract

**Objective:**

To assess the associations between neonatal hyperglycaemia and insulin treatment, versus long-term neurodevelopmental outcomes in children born extremely preterm.

**Design and setting:**

Observational national cohort study (Extremely Preterm Infants in Sweden Study) using prospectively and retrospectively collected data. Neurodevelopmental assessment was performed at 6.5 years of age.

**Patients:**

533 infants born <27 gestational weeks during 2004–2007; 436 survivors were assessed at 6.5 years.

**Outcome measures:**

Neurodevelopmental disability (NDD), survival without moderate to severe NDD, Wechsler Intelligence Scale for Children IV Full scale intelligence quotient (WISC-IV FSIQ) and Movement Assessment Battery for Children 2 (MABC-2) total score.

**Results:**

Duration of neonatal hyperglycaemia >8 mmol/L was associated with WISC-IV scores—for each day with hyperglycaemia there was a decrease of 0.33 points (95% CI 0.03 to 0.62) in FSIQ. Neonatal hyperglycaemia >8 mmol/L occurring on 3 consecutive days was associated with lower MABC-2 scores (adjusted mean difference: −4.90; 95% CI −8.90 to −0.89). For each day with hyperglycaemia >8 mmol/L, there was a decrease of 0.55 points (95% CI 0.17 to 0.93) in MABC-2 total score. Insulin treatment was not associated with any of the outcome measures.

**Conclusion:**

Neonatal hyperglycaemia >8 mmol/L was associated with lower intelligence scores and worse motor outcomes at 6.5 years of age. Insulin treatment was not associated with either worsened or improved neurodevelopmental outcomes. Randomised controlled trials are needed to clarify the role of insulin in treating hyperglycaemia in extremely preterm infants.

**What is already known on this topic?**

- Extremely preterm infants are prone to neonatal hyperglycaemia.
- Neonatal hyperglycaemia has been associated with multiple morbidities and has been suggested to be associated with neurodevelopmental disability.
- Insulin treatment for neonatal hyperglycaemia has shown conflicting results.

**What this study adds?**

- Both magnitude and duration of neonatal hyperglycaemia were associated with worse motor outcomes at 6.5 years of age and might be associated with lower intelligence scores.
- These associations were observed at glucose concentrations >8 mmol/L.
- Insulin treatment was not associated with either worsened or improved neurodevelopmental outcomes at 6.5 years of age, but further studies are required.

## Introduction

Children and adolescents born extremely preterm (EPT) are at considerable risk of long-term health problems and often have special educational needs.^1 2^ Approximately 25% of children born EPT have moderate to severe disability at 2.5 years of age,^3^ and a third have motor impairments at 6.5–15 years of age.^4 5^

Neonatal hyperglycaemia is common in EPT infants, occurring in up to 80% of infants, and it has been associated with increased mortality and white matter reduction at term age.^6 7^ Furthermore, hyperglycaemia was shown to have a negative effect on brain activity during the first 3 days of life in very preterm (VPT) infants.^8^ However, conflicting results have been published so far regarding the association between hyperglycaemia and later neurodevelopmental outcomes in preterm infants.^9 10^

Insulin treatment is a treatment strategy for neonatal hyperglycaemia, aiming to improve the glucose utilisation of the preterm infant.^11^ The benefit of insulin treatment has been debated. Retrospective studies have reported conflicting results, from increased survival at postnatal days 28 and 70 in EPT infants^6^ to no effect and even increased mortality and neurodevelopmental disability (NDD) at 2 years of age in children born VPT.^10 12^ Randomised controlled studies have not shown a beneficial effect of insulin treatment on mortality and neurodevelopmental outcomes in different paediatric patient groups, including preterm infants.^13–16^

This study aimed at assessing the associations between neonatal hyperglycaemia and insulin treatment, and neurodevelopmental outcomes at 6.5 years of age in children born EPT. We hypothesised that neonatal hyperglycaemia would be associated with worse neurodevelopmental outcomes, while insulin treatment would be associated with better neurodevelopmental outcomes.

## Methods

### Study population

The Extremely Preterm Infants in Sweden Study (EXPRESS) includes all infants who were born at gestational age <27 weeks between 1 April 2004 and 31 March 2007 in Sweden. Cohort characteristics, perinatal data, glycaemic status data, nutritional intakes, survival, morbidity and neurodevelopmental outcomes at age 6.5 years have been previously published.^4 6 17–19^

**Figure 1:**
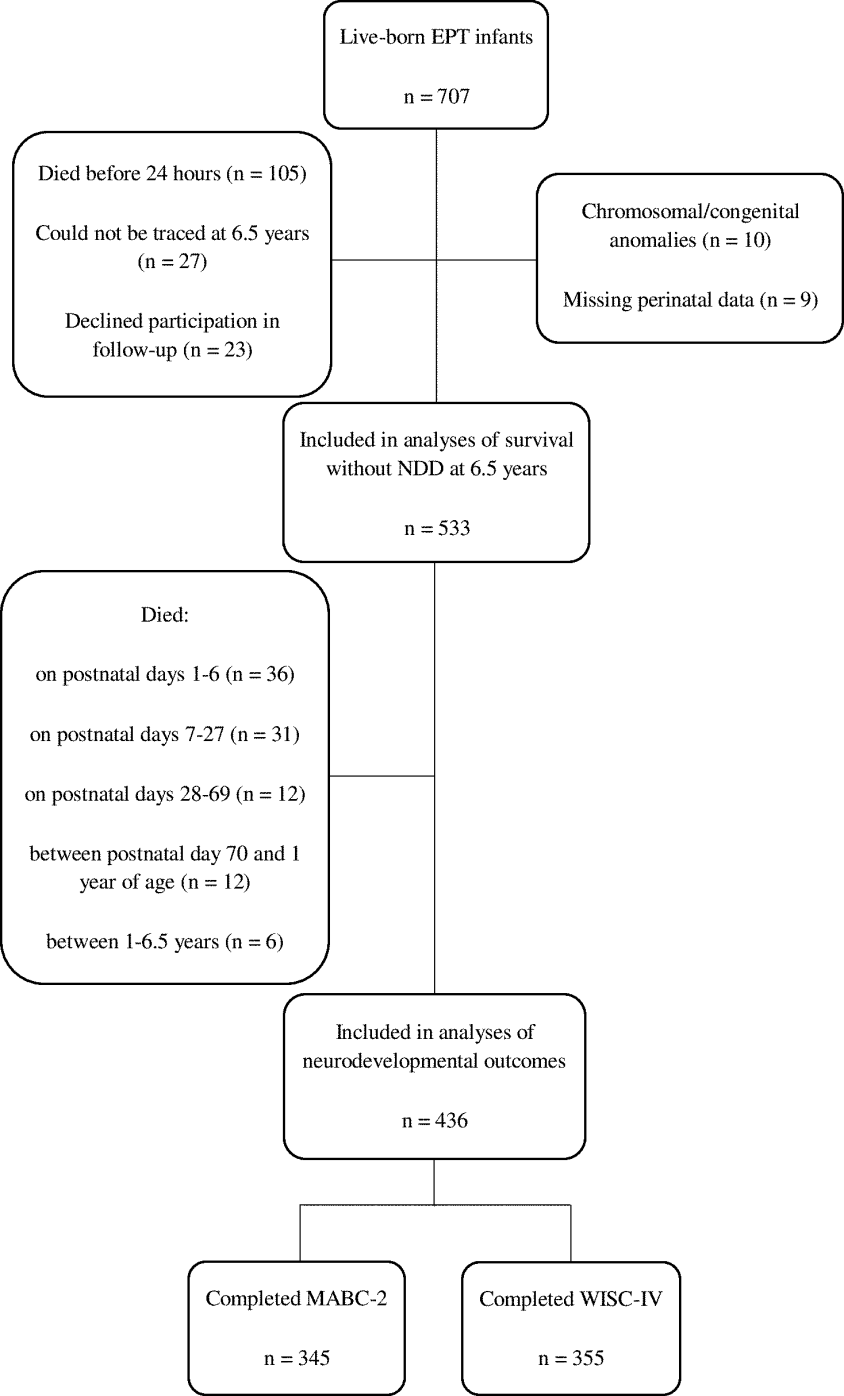
Study flow chart. EPT, extremely preterm; MABC-2, Movement Assessment Battery for Children 2; NDD, neurodevelopmental disability; WISC-IV, Wechsler Intelligence Scale for Children IV.

Of 707 live-born infants, 602 survived the first 24 hours of life and 533 (88.5%) of those were included in this study (figure 1). Eleven and 23 infants died before reaching 48 and 72 hours of age, respectively. These infants were excluded from analyses involving neonatal hyperglycaemia occurring on 2 and 3 consecutive days (and insulin treatment in infants exposed to such hyperglycaemia), respectively. Ninety-seven children died before the age of 6.5 years (figure 1), and thus, 436 (72.4%) of the children who survived the first 24 hours of life were included in analyses regarding neurodevelopmental outcomes. It was previously shown that the perinatal and sociodemographic characteristics and disability rates in the follow-up group did not differ from those who were not followed-up.^17^

### Perinatal and glycaemic status data

Prospectively collected perinatal data was retrieved from the EXPRESS database. Glycaemic status data collection was performed as previously described.^6^ Briefly, all glucose measurements and insulin treatment data available during the first 28 postnatal days were retrospectively collected from hospital records. For each infant, daily highest and lowest glucose concentrations were registered for the first 28 postnatal days, and the respective means were calculated. As no clear definition of hyperglycaemia in this patient population exists, several prospectively defined definitions were used: a highest glucose measurement >8, >10, >12 or >14 mmol/L, occurring at least once or on 2 or 3 consecutive days during the first 28 postnatal days (12 different definitions in total). Insulin treatment was given according to the clinical judgement of the attending physician, since no uniform national guideline for insulin treatment of preterm infants existed in Sweden during the study period.

### Neurodevelopmental outcomes

Prospectively collected data from a neurodevelopmental assessment performed at 6.5 years of age at the seven university hospitals in Sweden was retrieved from the EXPRESS database.^17^ Wechsler Intelligence Scale for Children IV (WISC-IV; n=355) was used to measure cognitive ability and was administered by psychologists.^20^ Movement Assessment Battery for Children 2 (MABC-2; n=345), a standardised test assessing gross and fine motor function (manual dexterity, balance, ball skills), was administered by physiotherapists.^21^ MABC-2 total scores ≤75 and ≤68 are equivalent to ≤15th centile (borderline motor impairment) and ≤5th centile (developmental coordination disorder) in Swedish children, respectively.^4^ The Gross Motor Function Classification System (GMFCS) was used to assess functional level in children with cerebral palsy.^22^ Hearing assessment was based on the child’s need of hearing aids. Visual acuity was assessed by ophthalmologists and classified according to modified WHO criteria. Data regarding maternal education was collected using a parental questionnaire.

Children not tested with WISC-IV but who had been physically examined received an NDD category according to clinical examination and medical record review (n=11). Medical record review was also used to assign an NDD category to children who were not physically examined (n=59).

The NDD categories were previously defined in the EXPRESS cohort as follows: no or mild NDD—having Full Scale Intelligence Quotient (FSIQ) score ≥−2 SD, no/mild cognitive disability on clinical examination or medical record review, no cerebral palsy or cerebral palsy (GMFCS 1), and visual acuity of ≥20/63 in the better eye; moderate to severe NDD—having FSIQ score <−2 SD or moderate/severe cognitive disability on clinical examination or medical record review, or cerebral palsy (GMFCS ≥2), or visual acuity of <20/63 in the better eye, or hearing impairment necessitating the use of hearing aid.^17^

### Statistical analyses

The analyses were done using SPSS Statistical software (V.26.0; IBM, Armonk, New York, USA). Possible associations were assessed between neonatal hyperglycaemia (yes/no), total duration of neonatal hyperglycaemia (in days), insulin treatment in hyperglycaemic infants (yes/no) and the following outcomes at 6.5 years of age: NDD (no or mild vs moderate to severe), survival without moderate to severe NDD (vs death or survival with moderate to severe NDD), WISC-IV scores (FSIQ scores) and MABC-2 total scores. The associations were evaluated using generalised linear mixed models with an appropriate linear or logistic link function and with a random intercept to account for clustering effect of twins/triplets. Furthermore, in analyses involving insulin treatment, clustering effect of treating hospital was considered in order to account for clinical practice variation regarding insulin treatment.

Possible confounders were identified using directed acyclic graphs. In multivariable models assessing the exposure of hyperglycaemia, the following possible confounders were considered a priori: gestational age at birth, sex, occurrence of intraventricular haemorrhage grade 3–4 and/or periventricular leukomalacia, and educational status of the mother. Furthermore, the following were considered for the first 14 postnatal days: durations of mechanical ventilation and steroid treatments, surgery due to patent ductus arteriosus or necrotising enterocolitis and number of culture-verified sepsis episodes. Models assessing the exposure of insulin treatment were adjusted for the duration of hyperglycaemia (in days). These models were in a later stage further adjusted for clinical characteristics that differed between infants treated and not-treated with insulin. Furthermore, all models including MABC-2 scores were adjusted for age at follow-up. Children with missing data in a certain variable were excluded from analyses including that specific variable. The significance level was set to p<0.05.

## Results

Cohort characteristics are described in table 1. Infants exposed to neonatal hyperglycaemia >10 mmol/L were more often males, had lower gestational age and weight z-score at birth, higher Clinical Risk Index for Babies (CRIB) score and were treated with steroids and mechanical ventilation for longer periods, and insulin-treated infants had lower gestational age at birth and higher prevalence of perinatal morbidities than non-treated infants with hyperglycaemia (table 1). Short-term morbidity and 1-year survival data, as well as follow-up data at 6.5 years of age, including neurodevelopmental outcomes, were previously published.^4 17 18^ Briefly, 33.6% of the children had moderate to severe NDD at 6.5 years of age. Mean (±SD) FSIQ score was 83.4 (14.8), developmental coordination disorder was found in 22.2% and borderline motor impairment was found in further 13.1% of the children. Children tested with WISC-IV and MABC-2 did not differ in their baseline characteristics from children not tested with either of these tests (results not shown), except for a longer duration of mechanical ventilation treatment in non-tested children (7.3 vs 6.0 days for both tests; p=0.042 for WISC-IV and 0.015 for MABC-2).

**Table 1:** Cohort characteristics

Characteristic	The entire cohort (n=533)	No hyperglycaemia (n=164)	Hyperglycaemia (n=369)	P value*	Hyperglycaemic infants		P value
					No insulin (n=298)	Insulin (n=71)	
**Perinatal variables**							
Gestational age, weeks, mean (±SD)	25.3 (1.1)	25.9 (0.8)	25.1 (1.1)	<0.001	25.2 (1.1)	24.5 (0.9)	<0.001
Birth weight z-score, mean (±SD)	−0.80 (1.2)	−0.58 (1.1)	−0.90 (1.3)	0.005	−0.89 (1.3)	−0.95 (1.3)	0.717
Male sex, n (%)	288 (54.0)	77 (47.0)	211 (57.2)	0.031	171 (57.4)	40 (56.3)	0.894
Singleton birth, n (%)	417 (78.2)	131 (79.9)	286 (77.5)	0.571	233 (78.2)	53 (74.6)	0.529
CRIB score, mean (±SD)	6.5 (3.7)	5.1 (3.7)	7.1 (3.5)	<0.001	6.7 (3.5)	8.7 (3.2)	<0.001
Antenatal steroid treatment, mean no. of doses (±SD)	1.5 (0.7)	1.6 (0.7)	1.5 (0.7)	0.592	1.5 (0.7)	1.7 (0.6)	0.045
**Neonatal variables**							
Days with mechanical ventilation treatment, median (range)†	5 (0–14)	2 (0–14)	7 (0–14)	<0.001	7 (0–14)	11 (0–14)	<0.001
Days with steroid treatment, mean (±SD)†	0.3 (1.5)	0.1 (0.9)	0.4 (1.7)	0.007	0.3 (1.4)	1.1 (2.3)	0.008
Sepsis episodes, median (range)†	0 (0–3)	0 (0–2)	0 (0–3)	0.467	0 (0–3)	0 (0–2)	0.959
IVH grade 3–4 and/or PVL, n (%)	95 (17.8)	29 (17.7)	66 (17.9)	1.000	52 (17.4)	14 (19.7)	0.730
Surgically-treated NEC, n (%)†	11 (2.1)	3 (1.8)	8 (2.2)	1.000	4 (1.3)	4 (5.6)	0.048
Surgically-treated PDA, n (%)†	31 (5.8)	5 (3.0)	26 (7.0)	0.074	13 (4.4)	13 (18.3)	<0.001
Daily highest glucose concentration, mmol/L, mean (±SD)‡	7.9 (2.3)	5.8 (0.9)	8.7 (2.2)	<0.001	8.2 (1.8)	10.8 (2.6)	<0.001
Daily lowest glucose concentration, mmol/L, mean (±SD)‡	5.6 (1.4)	4.7 (1.0)	6.0 (1.4)	<0.001	5.9 (1.3)	6.5 (1.6)	0.003
Days with hyperglycaemia, mean (±SD)‡	3.7 (4.9)	0 (0)	5.3 (5.2)	<0.001	3.8 (3.5)	11.3 (6.6)	<0.001
**Socioeconomic variables**							
Education status of the mother, n (%)§	(n=405)	(n=127)	(n=278)		(n=224)	(n=54)	
≤9 years	38 (9.4)	8 (6.3)	30 (10.8)	0.041¶	23 (10.3)	7 (13.0)	0.588¶
10–11 years	26 (6.4)	12 (9.4)	14 (5.0)		13 (5.8)	1 (1.9)	
12–13 years	141 (34.8)	35 (27.6)	106 (38.1)		87 (38.8)	19 (35.2)	
14–15 years	100 (24.7)	32 (25.2)	68 (24.5)		51 (22.8)	17 (31.5)	
16 years	45 (11.1)	16 (12.6)	29 (10.4)		25 (11.2)	4 (7.4)	
≥17 years	55 (13.6)	24 (18.9)	31 (11.2)		25 (11.2)	6 (11.1)	
Non-Nordic origin of mother, n (%)	88 (20.2) (n=436)	16 (11.6) (n=138)	72 (24.2) (n=298)	0.002	63 (26.1) (n=241)	9 (15.8) (n=57)	0.101

Hyperglycaemia—glucose concentration measurement >10 mmol/L at least once during the first 28 postnatal days; Student’s t-test and χ^2^ test were used as appropriate.

*P value for comparison between infants with and without neonatal hyperglycaemia.

†During the first 14 postnatal days.

‡During the first 28 postnatal days.

§Percentage based on known values.

¶P value for overall difference between groups with available data.

CRIB, Clinical Risk Index for Babies; IVH, intraventricular haemorrhage; NEC, necrotising enterocolitis; PDA, patent ductus arteriosus; PVL, periventricular leukomalacia.

### NDD at 6.5 years of age

Neonatal hyperglycaemia and insulin treatment were not significantly associated with moderate to severe NDD at 6.5 years of age in adjusted models, regardless of hyperglycaemia definition or duration (table 2 and online supplemental tables 1 and 2).

10.1136/archdischild-2020-319926.supp1 Supplementary data

10.1136/archdischild-2020-319926.supp2 Supplementary data

**Table 2:** Univariable and multivariable generalised linear mixed models for the associations between neonatal hyperglycaemia (>8 and >10 mmol/L) and moderate to severe neurodevelopmental disability at 6.5 years of age in children born extremely preterm

Hyperglycaemia definition		Moderate to severe NDD		Crude OR (95% CI)	P value	aOR (95% CI)	P value
		No hyperglycaemia	Hyperglycaemia				
>8 mmol/L	at least once	12/56 (21.4%)	132/380 (34.7%)	1.95 (0.99 to 3.84)	0.055	1.27 (0.60 to 2.67)	0.532
	on ≥2 consecutive days	40/160 (25.0%)	104/276 (37.7%)	1.81 (1.17 to 2.81)	0.008	1.16 (0.68 to 1.97)	0.588
	on ≥3 consecutive days	68/252 (27.0%)	76/184 (41.3%)	1.90 (1.26 to 2.87)	0.002	1.36 (0.83 to 2.24)	0.226
	Days with hyperglycaemia (per 1-day increase)	–	–	1.07 (1.04 to 1.10)	<0.001	1.03 (0.99 to 1.08)	0.176
>10 mmol/L	at least once	37/138 (26.8%)	107/298 (35.9%)	1.53 (0.97 to 2.40)	0.066	1.08 (0.63 to 1.87)	0.775
	on ≥2 consecutive days	76/251 (30.3%)	68/185 (36.8%)	1.34 (0.89 to 2.01)	0.165	0.88 (0.53 to 1.48)	0.637
	on ≥3 consecutive days	91/319 (28.5%)	53/117 (45.3%)	2.07 (1.33 to 3.23)	0.001	1.42 (0.82 to 2.45)	0.214
	Days with hyperglycaemia (per 1-day increase)	–	–	1.08 (1.04 to 1.13)	<0.001	1.04 (0.98 to 1.10)	0.178

A random intercept was used to account for the clustering effect of twins/triplets. Adjusted for gestational age at birth, sex, occurrence of intraventricular haemorrhage grade 3–4 and/or periventricular leukomalacia, durations of mechanical ventilation and steroid treatments during the first 14 postnatal days, surgery due to patent ductus arteriosus or necrotising enterocolitis during the first 14 postnatal days, number of culture-verified sepsis episodes during the first 14 postnatal days and educational status of the mother.

NDD, neurodevelopmental disability.

### Survival without moderate to severe NDD at 6.5 years of age

Neonatal hyperglycaemia and insulin treatment were not significantly associated with survival without moderate to severe NDD at 6.5 years of age in adjusted models, regardless of hyperglycaemia definition or duration (table 3 and online supplemental tables 3 and 4).

10.1136/archdischild-2020-319926.supp3 Supplementary data

10.1136/archdischild-2020-319926.supp4 Supplementary data

**Table 3:** Univariable and multivariable generalised linear mixed models for the associations between neonatal hyperglycaemia (>8 and >10 mmol/L) and survival without moderate to severe neurodevelopmental disability at 6.5 years of age in children born extremely preterm

Hyperglycaemia definition		Survival without moderate to severe NDD		Crude OR (95% CI)	P value	aOR (95% CI)	P value
		No hyperglycaemia	Hyperglycaemia				
>8 mmol/L	at least once	44/73 (60.3%)	249/460 (54.1%)	0.78 (0.46 to 1.31)	0.351	0.87 (0.49 to 1.55)	0.628
	on ≥2 consecutive days	120/184 (65.2%)	173/338 (51.2%)	0.56 (0.38 to 0.82)	0.003	1.16 (0.74 to 1.82)	0.517
	on ≥3 consecutive days	185/281 (65.8%)	108/229 (47.2%)	0.46 (0.32 to 0.67)	<0.001	1.54 (0.99 to 2.38)	0.056
	Days with hyperglycaemia (per 1-day increase)	–	–	0.96 (0.93 to 0.99)	0.003	1.00 (0.96 to 1.04)	0.965
>10 mmol/L	at least once	102/164 (62.2%)	191/369 (51.8%)	0.66 (0.45 to 0.96)	0.034	1.13 (0.71 to 1.78)	0.606
	on ≥2 consecutive days	176/291 (60.5%)	117/231 (50.6%)	0.67 (0.47 to 0.96)	0.031	1.03 (0.66 to 1.59)	0.913
	on ≥3 consecutive days	229/362 (63.3%)	64/148 (43.2%)	0.44 (0.30 to 0.66)	<0.001	1.47 (0.91 to 2.39)	0.115
	Days with hyperglycaemia (per 1-day increase)	–	–	0.94 (0.90 to 0.97)	0.001	1.02 (0.97 to 1.07)	0.400

A random intercept was used to account for the clustering effect of twins/triplets. Adjusted for gestational age at birth, sex, occurrence of intraventricular haemorrhage grade 3–4 and/or periventricular leukomalacia, durations of mechanical ventilation and steroid treatments during the first 14 postnatal days, surgery due to patent ductus arteriosus or necrotising enterocolitis during the first 14 postnatal days, number of culture-verified sepsis episodes during the first 14 postnatal days and educational status of the mother.

NDD, neurodevelopmental disability.

### WISC-IV scores

Neonatal hyperglycaemia >8 mmol/L occurring on 2 consecutive days (but not according to other hyperglycaemia definitions) was significantly associated with lower WISC-IV scores at 6.5 years of age in adjusted models (table 4 and online supplemental table 5). A longer total duration of exposure to neonatal hyperglycaemia >8 mmol/L (but not according to other hyperglycaemia definitions) was significantly associated with lower WISC-IV scores. Insulin treatment was not significantly associated with WISC-IV scores, regardless of hyperglycaemia definition or duration (except for hyperglycaemia >14 mmol/L occurring on 3 consecutive days; online supplemental table 6).

10.1136/archdischild-2020-319926.supp5 Supplementary data

10.1136/archdischild-2020-319926.supp6 Supplementary data

**Table 4:** Univariable and multivariable generalised linear mixed models for the associations between neonatal hyperglycaemia (>8 and >10 mmol/L) and Wechsler Intelligence Scale for Children IV (WISC-IV) score at 6.5 years of age in children born extremely preterm

Hyperglycaemia definition		Mean WISC-IV score		Univariable		Multivariable	
		No hyperglycaemia (n)	Hyperglycaemia (n)	Difference in mean scores (95% CI)	P value	Difference in mean scores (95% CI)	P value
>8 mmol/L	at least once	88.82 (45)	83.28 (310)	−5.53 (−9.90 to −1.16)	0.013	−2.58 (−6.79 to 1.64)	0.230
	on ≥2 consecutive days	88.14 (130)	81.6 (225)	−6.51 (−9.49 to −3.53)	<0.001	−3.17 (−6.26 to −0.08)	0.045
	on ≥3 consecutive days	86.42 (208)	80.63 (147)	−5.79 (−8.77 to −2.80)	<0.001	−2.58 (−5.66 to 0.49)	0.099
	Days with hyperglycaemia (per 1-day increase)	–	–	−0.66 (−0.90 to −0.42)	<0.001	−0.33 (−0.62 to −0.03)	0.029
>10 mmol/L	at least once	86.75 (114)	82.71 (241)	−4.04 (−7.23 to −0.84)	0.013	−1.57 (−4.79 to 1.65)	0.337
	on ≥2 consecutive days	85.78 (208)	81.53 (147)	−4.25 (−7.29 to −1.21)	0.006	−1.06 (−4.21 to 2.09)	0.510
	on ≥3 consecutive days	85.65 (263)	79.36 (92)	−6.29 (−9.69 to −2.89)	<0.001	−2.24 (−5.83 to 1.35)	0.220
	Days with hyperglycaemia (per 1-day increase)	–	–	−0.72 (−1.05 to −0.39)	<0.001	−0.31 (−0.69 to 0.08)	0.117

A random intercept was used to account for the clustering effect of twins/triplets. Adjusted for gestational age at birth, sex, occurrence of intraventricular haemorrhage grade 3–4 and/or periventricular leukomalacia, durations of mechanical ventilation and steroid treatments during the first 14 postnatal days, surgery due to patent ductus arteriosus or necrotising enterocolitis during the first 14 postnatal days, number of culture-verified sepsis episodes during the first 14 postnatal days and educational status of the mother.

### MABC-2 scores

Neonatal hyperglycaemia >10, >12 and >14 mmol/L, regardless of frequency of occurrence (except for hyperglycaemia >12 mmol/L occurring on 3 consecutive days and hyperglycaemia >14 mmol/L occurring on 2 consecutive days), as well as hyperglycaemia >8 mmol/L occurring on 3 consecutive days, were significantly associated with lower MABC-2 scores at 6.5 years of age in adjusted models (Cohen’s d<0.2; table 5 and online supplemental table 7). A longer total duration of exposure to neonatal hyperglycaemia was significantly associated with lower MABC-2 scores, regardless of hyperglycaemia definition. Insulin treatment was not significantly associated with MABC-2 scores, regardless of hyperglycaemia definition or duration (online supplemental table 8).

10.1136/archdischild-2020-319926.supp7 Supplementary data

10.1136/archdischild-2020-319926.supp8 Supplementary data

**Table 5:** Univariable and multivariable generalised linear mixed models for the associations between neonatal hyperglycaemia (>8 and >10 mmol/L) and Motor Assessment Battery for Children 2 (MABC-2) score at 6.5 years of age in children born extremely preterm

Hyperglycaemia definition		Mean MABC-2 score		Univariable		Multivariable	
		No hyperglycaemia (n)	Hyperglycaemia (n)	Difference in mean scores (95% CI)	P value	Difference in mean scores (95% CI)	P value
>8 mmol/L	at least once	73.49 (44)	64.55 (301)	−8.94 (−14.67 to −3.22)	0.002	−4.73 (−10.20 to 0.74)	0.090
	on ≥2 consecutive days	70.27 (129)	62.99 (216)	−7.27 (−11.19 to −3.36)	<0.001	−3.24 (−7.26 to 0.79)	0.114
	on ≥3 consecutive days	68.88 (204)	61.09 (141)	−7.79 (−11.69 to −3.89)	<0.001	−4.90 (−8.90 to −0.89)	0.017
	Days with hyperglycaemia (per 1-day increase)	–	–	−0.84 (−1.15 to −0.53)	<0.001	−0.55 (−0.93 to −0.17)	0.005
>10 mmol/L	at least once	70.91 (114)	63.16 (231)	−7.75 (−11.83 to −3.68)	<0.001	−4.64 (−8.78 to −0.51)	0.029
	on ≥2 consecutive days	69.48 (203)	60.31 (142)	−9.17 (−13.04 to −5.29)	<0.001	−5.87 (−9.94 to −1.79)	0.005
	on ≥3 consecutive days	68.17 (256)	58.48 (89)	−9.69 (−14.09 to −5.29)	<0.001	−6.05 (−10.71 to −1.39)	0.011
	Days with hyperglycaemia (per 1-day increase)	–	–	−1.18 (−1.59 to −0.77)	<0.001	−0.91 (−1.39 to −0.42)	<0.001

A random intercept was used to account for the clustering effect of twins/triplets. Adjusted for gestational age at birth, sex, occurrence of intraventricular haemorrhage grade 3–4 and/or periventricular leukomalacia, durations of mechanical ventilation and steroid treatments during the first 14 postnatal days, surgery due to patent ductus arteriosus or necrotising enterocolitis during the first 14 postnatal days, number of culture-verified sepsis episodes during the first 14 postnatal days, educational status of the mother, and age at follow-up. MABC-2 total scores ≤75 and ≤68 are equivalent to ≤15th centile (borderline motor impairment) and ≤5th centile (developmental coordination disorder) in Swedish children, respectively.

## Discussion

In this cohort of EPT infants, we observed that neonatal hyperglycaemia and its duration were associated with lower MABC-2 scores but not with NDD at 6.5 years of age, adjusted for multiple clinical factors. Significant associations with worse motor outcomes were observed when using hyperglycaemia threshold definition as low as >8 mmol/L. Hyperglycaemia was also associated with lower WISC-IV scores. Insulin treatment was not associated with either worsened or improved neurodevelopmental outcomes at 6.5 years of age.

We report a novel finding of an association between neonatal hyperglycaemia and lower MABC-2 scores. Significant associations were found for exposure to hyperglycaemia >10, >12 and >14 mmol/L of almost all frequency definitions, and only for hyperglycaemia >8 mmol/L occurring on 3 consecutive days. These findings suggest a possible dose–response relationship between glucose concentrations and the outcome. For example, for each additional day with hyperglycaemia >8 mmol/L, there was observed a decrease of MABC-2 score by 0.55 points, while for each additional day with hyperglycaemia >14 mmol/L there was observed a decrease of MABC-2 score by 1.47 points. These findings might be of clinical importance since developmental coordination disorder is associated with poor health outcomes.^23^

It was previously shown that neonatal hyperglycaemia was not associated with neonatal illness or NDD at 2 years of age in children born VPT.^9^ However, the cohort in that study had higher mean gestational age compared with our cohort and neonatal hyperglycaemia was defined differently. The Hyperglycaemia In Neonates Trial (HINT) assessed neurocognitive function at 7 years of age in preterm-born children with neonatal hyperglycaemia, randomised to receive either standard or tight glycaemic control.^16^ No associations between glucose concentrations and MABC-2 scores were found, yet all infants included in that trial were hyperglycaemic while we compared hyperglycaemic and non-hyperglycaemic infants.

It has been suggested that preterm neonates exhibit both insulin resistance and impaired insulin production.^24^ Conflicting results have been published regarding the effect of insulin treatment for neonatal hyperglycaemia on neurodevelopmental outcomes. A retrospective study in preterm infants did not observe an association between insulin treatment and neurodevelopmental outcomes at 1 year of age.^12^ Infants included in that study had higher gestational age at birth, CRIB score and incidence of perinatal morbidities than our cohort. Another retrospective case–control study in children born VPT observed that infants treated with insulin for neonatal hyperglycaemia (after not responding to glucose intake reduction) had a higher incidence of abnormal neurological development at 2 years of age compared with infants not exposed to hyperglycaemia.^10^ In the HINT study, infants who had glucose concentrations in the range 4–6 mmol/L were more likely to survive without NDD and low WISC-IV scores, yet tight glycaemic control was not associated with a difference in NDD, survival without NDD, WISC-IV or MABC-2 scores at 7 years of age.^16^ In our study, a slightly different definition for NDD was used (lower FSIQ score, higher visual acuity, MABC-2 not a criteria) and different confounders were adjusted for. Furthermore, infants treated with insulin included in our study were generally sicker than hyperglycaemic infants not treated with insulin. Despite this, no differences were found in the studied outcomes, which might suggest a potential positive effect of insulin treatment. Further adjusting for variables differing between infants treated and not-treated with insulin did not affect the results. However, no definite conclusion regarding such a positive effect can be drawn from the current observational study design and further intervention studies are needed to delineate the long-term effects of insulin treatment.

The association between neonatal hyperglycaemia and lower WISC-IV scores in comparison with non-hyperglycaemic infants is a novel finding. However, we observed this association only for hyperglycaemia >8 mmol/L and not when higher thresholds were used. This might be due to limited statistical power or a chance finding. Nevertheless, the results call for further investigation regarding the association between neonatal hyperglycaemia and intelligence outcomes.

The mechanisms behind the possible effect of hyperglycaemia on neurocognitive functions are unclear. Lower brain weight and more severe brain damage, especially in the hippocampus region, were observed in hyperglycaemic rats.^25^ Furthermore, neonatal hyperglycaemia was found to induce oxidative stress, apoptosis and an increase in inflammatory cytokines, with ensuing microglial activation and astrocytosis.^26^ These changes resulted in long-term changes in synaptogenesis and behaviour in rats. Higher methylation of leucine-rich alpha-2-glycoprotein 1 (a protein associated with neurodevelopment), as well as upregulation of poly (ADP-ribose) polymerase-1 and NF-kB expression (associated with microglial activation) were associated with higher glucose concentrations.^27 28^ In EPT infants, hyperglycaemia >8.3 mmol/L on the first day of life was associated with white matter reduction at term-equivalent age.^7^ A meta-analysis concluded that neonatal hypoglycaemia was associated with visual-motor impairments, thus implying a possible effect of hyperglycaemia on motor development.^29^

To the best of our knowledge, our study is the largest study conducted in EPT infants investigating the associations between neonatal hyperglycaemia and neurodevelopmental outcomes in childhood. Other strengths of this study include its prospective design regarding neurodevelopmental outcomes data and the broad consideration of possible confounders. A limitation of the study is its retrospective design regarding glycaemic status data. Hospitals differed significantly in the frequency of glucose sampling; however, we have previously shown that no site-dependent differences in hyperglycaemia prevalence were found in this cohort.^6^ Insulin treatment might have had an impact on the magnitude and duration of hyperglycaemia and vice versa. Insulin treatment might not have had a sufficient effect on glucose concentrations or was introduced late, as reflected by the longer duration of exposure to hyperglycaemia in insulin-treated infants (table 1). However, the effect of insulin treatment on glucose concentrations could not be assessed, and thus, a definite conclusion regarding the effectiveness of insulin treatment cannot be drawn. Furthermore, as this was an observational study, our results represent associations and not necessarily causal effects. Although potential confounders have been considered, residual confounding cannot be excluded. The effect size of hyperglycaemia on neurodevelopmental outcomes was relatively small and this study was not originally designed and powered to measure the associations reported.

In conclusion, neonatal hyperglycaemia >8 mmol/L and longer duration of hyperglycaemia were associated with lower intelligence scores and worse motor outcomes at 6.5 years of age in children born EPT. Insulin treatment in hyperglycaemic infants was not associated with neurodevelopmental outcomes at 6.5 years of age. This observational study highlights the potential detrimental long-term effects of neonatal hyperglycaemia, while the benefit of insulin treatment remains unclear. Further studies, including adequately powered randomised controlled trials, are needed in order to better define neonatal hyperglycaemia in preterm infants, to guide its treatment and clarify its consequences.
