# Supplementary material for: Neonatal hyperglycaemia is associated with worse neurodevelopmental outcomes in extremely preterm infants
Source: Arch Dis Child Fetal Neonatal Ed. 2021 Apr 16;106(5):460–6. doi: 10.1136/archdischild-2020-319926 (PMC8394751; doi:10.1136/archdischild-2020-319926)
Supplement: Supplementary data [file archdischild-2020-319926supp001.pdf]

Online supplementary table 1. Univariable and multivariable generalised linear mixed models for the associations between neonatal hyperglycaemia (>12 and >14 mmol/L) and moderate to severe neurodevelopmental disability at 6.5 years of age in children born extremely preterm.

**Hyperglycaemia definition**		**Moderate to severe NDD**		**Crude OR (95% CI)**	**P value**	**aOR (95% CI)**	**P value**
		**No hyperglycaemia**	**Hyperglycaemia**				
**>12 mmol/L**	at least once	60/213 (28.2%)	84/223 (37.7%)	1.54 (1.02 to 2.31)	0.039	1.03 (0.63 to 1.70)	0.896
	on ≥2 consecutive days	97/314 (30.9%)	47/122 (38.5%)	1.40 (0.90 to 2.18)	0.139	0.93 (0.55 to 1.58)	0.796
	on ≥3 consecutive days	114/375 (30.4%)	30/61 (49.2%)	2.21 (1.27 to 3.84)	0.005	1.27 (0.66 to 2.44)	0.479
	Days with hyperglycaemia (per 1 day increase)	-	-	1.09 (1.03 to 1.15)	0.002	1.02 (0.95 to 1.10)	0.547
**>14 mmol/L**	at least once	79/267 (29.6%)	65/169 (38.5%)	1.48 (0.98 to 2.24)	0.061	1.02 (0.62 to 1.70)	0.927
	on ≥2 consecutive days	114/362 (31.5%)	30/74 (40.5%)	1.48 (0.88 to 2.50)	0.141	0.83 (0.44 to 1.54)	0.547
	on ≥3 consecutive days	127/405 (31.4%)	17/31 (54.8%)	2.64 (1.25 to 5.58)	0.011	1.51 (0.62 to 3.65)	0.361

Hyperglycaemia definition	Moderate to severe NDD		Crude OR (95% CI)	P value	aOR (95% CI)	P value
	No hyperglycaemia	Hyperglycaemia				
Days with hyperglycaemia (per 1 day increase)	-	-	1.12 (1.04 to 1.20)	0.004	1.03 (0.94 to 1.13)	0.496

A random intercept was used to account for the clustering effect of twins/triplets. Adjusted for gestational age at birth, sex, occurrence of intraventricular haemorrhage grade 3-4 and/or periventricular leukomalacia, durations of mechanical ventilation and steroid treatments during the first 14 postnatal days, surgery due to patent ductus arteriosus or necrotising enterocolitis during the first 14 postnatal days, number of culture-verified sepsis episodes during the first 14 postnatal days, and educational status of the mother.
