# Supplementary material for: Neonatal hyperglycaemia is associated with worse neurodevelopmental outcomes in extremely preterm infants
Source: Arch Dis Child Fetal Neonatal Ed. 2021 Apr 16;106(5):460–6. doi: 10.1136/archdischild-2020-319926 (PMC8394751; doi:10.1136/archdischild-2020-319926)
Supplement: Supplementary data [file archdischild-2020-319926supp002.pdf]

Online supplementary table 2. Univariable and multivariable generalised linear mixed models for the associations between insulin treatment in hyperglycaemic infants and moderate to severe neurodevelopmental disability at 6.5 years of age in children born extremely preterm.

**Hyperglycaemia definition**		**Moderate to severe NDD in hyperglycaemic infants**		**Crude OR (95% CI)**	**P value**	**aOR* (95% CI)**	**P value**	**aOR† (95% CI)**	**P value**
		**No insulin**	**Insulin**						
**>8 mmol/L**	at least once	102/323 (31.6%)	30/57 (52.6%)	2.33 (1.31 to 4.17)	0.004	1.33 (0.65 to 2.73)	0.441	1.17 (0.56 to 2.47)	0.677
	on ≥2 consecutive days	74/219 (33.8%)	30/57 (52.6%)	1.86 (1.01 to 3.43)	0.048	1.15 (0.55 to 2.41)	0.706	1.03 (0.48 to 2.22)	0.937
	on ≥3 consecutive days	48/131 (36.6%)	28/53 (52.8%)	1.73 (0.88 to 3.40)	0.114	1.28 (0.58 to 2.81)	0.540	1.00 (0.43 to 2.32)	0.999
**>10 mmol/L**	at least once	77/241 (32.0%)	30/57 (52.6%)	2.22 (1.22 to 4.04)	0.009	1.21 (0.57 to 2.56)	0.616	1.10 (0.51 to 2.38)	0.803
	on ≥2 consecutive days	40/130 (30.8%)	28/55 (50.9%)	2.00 (1.02 to 3.94)	0.045	1.08 (0.48 to 2.41)	0.857	1.05 (0.46 to 2.37)	0.912
	on ≥3 consecutive days	27/70 (38.6%)	26/47 (55.3%)	1.97 (0.91 to 4.23)	0.084	1.37 (0.57 to 3.28)	0.479	1.12 (0.44 to 2.86)	0.812
**>12 mmol/L**	at least once	54/167 (32.3%)	30/56 (53.6%)	2.15 (1.14 to 4.07)	0.019	1.56 (0.72 to 3.39)	0.264	1.45 (0.66 to 3.20)	0.353
	on ≥2 consecutive days	22/72 (30.6%)	25/50 (50.0%)	1.81 (0.80 to 4.05)	0.151	1.11 (0.43 to 2.83)	0.834	1.01 (0.39 to 2.65)	0.983

Hyperglycaemia definition		Moderate to severe NDD in hyperglycaemic infants		Crude OR (95% CI)	P value	aOR* (95% CI)	P value	aOR† (95% CI)	P value
		No insulin	Insulin						
	on ≥3 consecutive days	11/26 (42.3%)	19/35 (54.3%)	1.61 (0.55 to 4.74)	0.380	1.24 (0.37 to 4.12)	0.724	0.97 (0.25 to 3.70)	0.958
>14 mmol/L	at least once	37/116 (31.9%)	28/53 (52.8%)	2.30 (1.16 to 4.58)	0.018	1.74 (0.80 to 3.80)	0.163	1.54 (0.68 to 3.48)	0.299
	on ≥2 consecutive days	13/39 (33.3%)	17/35 (48.6%)	1.78 (0.67 to 4.72)	0.246	1.15 (0.38 to 3.47)	0.809	1.00 (0.30 to 3.33)	0.999
	on ≥3 consecutive days	6/10 (60.0%)	11/21 (52.4%)	0.69 (0.13 to 3.65)	0.652	0.55 (0.08 to 3.62)	0.521	0.06 (0.002 to 2.50)	0.134

Random intercepts were used to account for the clustering effects of twins/triplets and treating hospital.

* Adjusted for duration of hyperglycaemia (according to respective definition) during the first 28 postnatal days.

† Adjusted for gestational age at birth, durations of mechanical ventilation and steroid treatments during the first 14 postnatal days, surgery due to patent ductus arteriosus or necrotising enterocolitis during the first 14 postnatal days, and duration of hyperglycaemia (according to respective definition) during the first 28 postnatal days.
