# Supplementary material for: Neonatal hyperglycaemia is associated with worse neurodevelopmental outcomes in extremely preterm infants
Source: Arch Dis Child Fetal Neonatal Ed. 2021 Apr 16;106(5):460–6. doi: 10.1136/archdischild-2020-319926 (PMC8394751; doi:10.1136/archdischild-2020-319926)
Supplement: Supplementary data [file archdischild-2020-319926supp004.pdf]

Online supplementary table 4. Univariable and multivariable generalised linear mixed models for the associations between insulin treatment in hyperglycaemic infants and survival without moderate to severe neurodevelopmental disability at 6.5 years of age in children born extremely preterm.

**Hyperglycaemia definition**		**Survival without moderate to severe NDD in hyperglycaemic infants**		**Crude OR (95% CI)**	**P value**	**aOR* (95% CI)**	**P value**	**aOR† (95% CI)**	**P value**
		**No insulin**	**Insulin**						
**>8 mmol/L**	at least once	222/389 (57.1%)	27/71 (38.0%)	0.47 (0.28 to 0.81)	0.006	1.56 (0.82 to 2.96)	0.177	1.53 (0.79 to 2.97)	0.203
	on ≥2 consecutive days	146/267 (54.7%)	27/71 (38.0%)	0.57 (0.33 to 1.01)	0.054	1.41 (0.73 to 2.73)	0.301	1.37 (0.70 to 2.69)	0.359
	on ≥3 consecutive days	83/168 (49.4%)	25/64 (39.1%)	0.72 (0.39 to 1.34)	0.297	1.29 (0.62 to 2.66)	0.497	1.21 (0.57 to 2.58)	0.621
**>10 mmol/L**	at least once	164/298 (55.0%)	27/71 (38.0%)	0.54 (0.31 to 0.93)	0.028	1.27 (0.65 to 2.49)	0.478	1.29 (0.65 to 2.55)	0.472
	on ≥2 consecutive days	90/164 (54.9%)	27/68 (39.7%)	0.63 (0.34 to 1.46)	0.135	1.03 (0.51 to 2.09)	0.933	1.10 (0.53 to 2.27)	0.807
	on ≥3 consecutive days	43/91 (47.3%)	21/57 (36.8%)	0.65 (0.33 to 1.31)	0.229	1.17 (0.51 to 2.66)	0.712	1.07 (0.45 to 2.53)	0.884
**>12 mmol/L**	at least once	113/209 (54.1%)	26/69 (37.7%)	0.60 (0.33 to 1.07)	0.084	1.23 (0.61 to 2.49)	0.566	1.24 (0.60 to 2.57)	0.554

Hyperglycaemia definition		Survival without moderate to severe NDD in hyperglycaemic infants		Crude OR (95% CI)	P value	aOR* (95% CI)	P value	aOR† (95% CI)	P value
		**No insulin**	**Insulin**						
	on ≥2 consecutive days	50/93 (53.8%)	25/61 (41.0%)	0.69 (0.34 to 1.40)	0.306	0.93 (0.41 to 2.10)	0.864	0.96 (0.42 to 2.22)	0.924
	on ≥3 consecutive days	15/37 (40.5%)	16/43 (37.2%)	0.87 (0.34 to 2.27)	0.775	0.95 (0.33 to 2.80)	0.931	0.92 (0.28 to 2.97)	0.883
	at least once	79/154 (51.3%)	25/64 (39.1%)	0.68 (0.36 to 1.29)	0.242	1.10 (0.54 to 2.26)	0.796	1.04 (0.50 to 2.18)	0.919
**>14 mmol/L**	on ≥2 consecutive days	26/51 (51.0%)	18/44 (40.9%)	0.79 (0.33 to 1.90)	0.600	0.93 (0.35 to 2.44)	0.873	0.96 (0.35 to 2.62)	0.929
	on ≥3 consecutive days	4/16 (25.0%)	10/25 (40.0%)	2.03 (0.46 to 8.95)	0.340	0.42 (0.08 to 2.20)	0.292	0.26 (0.03 to 2.03)	0.190

Random intercepts were used to account for the clustering effects of twins/triplets and treating hospital.

* Adjusted for duration of hyperglycaemia (according to respective definition) during the first 28 postnatal days.

† Adjusted for gestational age at birth, durations of mechanical ventilation and steroid treatments during the first 14 postnatal days, surgery due to patent ductus arteriosus or necrotising enterocolitis during the first 14 postnatal days, and duration of hyperglycaemia (according to respective definition) during the first 28 postnatal days.
