# Supplementary material for: Neonatal hyperglycaemia is associated with worse neurodevelopmental outcomes in extremely preterm infants
Source: Arch Dis Child Fetal Neonatal Ed. 2021 Apr 16;106(5):460–6. doi: 10.1136/archdischild-2020-319926 (PMC8394751; doi:10.1136/archdischild-2020-319926)
Supplement: Supplementary data [file archdischild-2020-319926supp005.pdf]

Online supplementary table 5. Univariable and multivariable generalised linear mixed models for the associations between neonatal hyperglycaemia (>12 and >14 mmol/L) and Wechsler Intelligence Scale for Children IV (WISC-IV) score at 6.5 years of age in children born extremely preterm.

Hyperglycaemia definition		Mean WISC-IV score		Univariable		Multivariable	
		**No hyperglycaemia (n)**	**Hyperglycaemia (n)**	**Difference in mean scores (95% CI)**	**P value**	**Difference in mean scores (95% CI)**	**P value**
**>12 mmol/L**	at least once	86.03 (177)	81.98 (178)	-4.05 (-7.04 to -1.06)	0.008	-1.07 (-4.10 to 1.96)	0.487
	on ≥2 consecutive days	85.24 (262)	80.50 (93)	-4.74 (-8.17 to -1.32)	0.007	-1.43 (-4.87 to 2.02)	0.417
	on ≥3 consecutive days	84.78 (314)	78.12 (41)	-6.66 (-11.21 to -2.11)	0.004	-2.07 (-6.57 to 2.44)	0.368
	Days with hyperglycaemia (per 1 day increase)	-	-	-0.78 (-1.24 to -0.32)	0.001	-0.22 (-0.72 to 0.28)	0.383
**>14 mmol/L**	at least once	86.14 (222)	80.45 (133)	-5.70 (-8.74 to -2.65)	<0.001	-2.88 (-5.97 to 0.20)	0.067
	on ≥2 consecutive days	84.91 (299)	79.12 (56)	-5.79 (-9.76 to -1.82)	0.004	-1.41 (-5.37 to 2.56)	0.486
	on ≥3 consecutive days	84.45 (334)	76.85 (21)	-7.60 (-13.81 to -1.40)	0.017	-4.13 (-10.21 to 1.96)	0.183

**Hyperglycaemia definition**	**Mean WISC-IV score**		**Univariable**		**Multivariable**	
	**No hyperglycaemia (n)**	**Hyperglycaemia (n)**	**Difference in mean scores (95% CI)**	**P value**	**Difference in mean scores (95% CI)**	**P value**
Days with hyperglycaemia (per 1 day increase)	-	-	-1.00 (-1.63 to -0.37)	0.002	-0.40 (-1.05 to 0.24)	0.222

A random intercept was used to account for the clustering effect of twins/triplets. Adjusted for gestational age at birth, sex, occurrence of intraventricular haemorrhage grade 3-4 and/or periventricular leukomalacia, durations of mechanical ventilation and steroid treatments during the first 14 postnatal days, surgery due to patent ductus arteriosus or necrotising enterocolitis during the first 14 postnatal days, number of culture-verified sepsis episodes during the first 14 postnatal days, and educational status of the mother.
