# Supplementary material for: Neonatal hyperglycaemia is associated with worse neurodevelopmental outcomes in extremely preterm infants
Source: Arch Dis Child Fetal Neonatal Ed. 2021 Apr 16;106(5):460–6. doi: 10.1136/archdischild-2020-319926 (PMC8394751; doi:10.1136/archdischild-2020-319926)
Supplement: Supplementary data [file archdischild-2020-319926supp006.pdf]

Online supplementary table 6. Univariable and multivariable generalised linear mixed models for the associations between insulin treatment in hyperglycaemic infants and Wechsler Intelligence Scale for Children IV (WISC-IV) score at 6.5 years of age in children born extremely preterm.

**Hyperglycaemia definition**		**Mean WISC-IV score**		**Univariable**		**Multivariable**			
		**No insulin (n)**	**Insulin (n)**	**Difference in mean scores (95% CI)**	**P value**	**Difference in mean scores* (95% CI)**	**P value**	**Difference in mean scores† (95% CI)**	**P value**
**>8 mmol/L**	at least once	84.03 (265)	77.61 (45)	-6.43 (-11.01 to -1.85)	0.006	-0.80 (-6.18 to 4.57)	0.769	0.36 (-4.94 to 5.66)	0.894
	on ≥2 consecutive days	81.94 (180)	77.86 (45)	-4.08 (-8.85 to 0.69)	0.093	-0.73 (-6.18 to 4.73)	0.793	0.33 (-5.07 to 5.74)	0.904
	on ≥3 consecutive days	81.51 (106)	77.29 (41)	-4.22 (-9.13 to 0.69)	0.091	-1.84 (-7.48 to 3.81)	0.521	-0.08 (-5.77 to 5.61)	0.978
**>10 mmol/L**	at least once	83.34 (196)	77.73 (45)	-5.61 (-10.28 to -0.95)	0.019	-1.48 (-7.09 to 4.12)	0.602	0.30 (-5.31 to 5.91)	0.916
	on ≥2 consecutive days	82.26 (103)	78.60 (44)	-3.66 (-8.88 to 1.56)	0.168	-1.13 (-6.90 to 4.63)	0.699	0.43 (-5.52 to 6.38)	0.887
	on ≥3 consecutive days	81.67 (55)	75.53 (37)	-6.14 (-12.52 to 0.24)	0.059	-4.33 (-11.42 to 2.77)	0.229	-1.68 (-9.13 to 5.77)	0.654
**>12 mmol/L**	at least once	82.72 (134)	77.41 (44)	-5.31 (-10.34 to -0.28)	0.039	-3.65 (-9.86 to 2.56)	0.248	-2.46 (-8.63 to 3.70)	0.431
	on ≥2 consecutive days	81.89 (54)	80.16 (39)	-1.73 (-7.29 to 3.84)	0.540	0.30 (-6.14 to 6.73)	0.928	1.50 (-5.28 to 8.29)	0.661

Hyperglycaemia definition		Mean WISC-IV score		Univariable		Multivariable			
		No insulin (n)	Insulin (n)	Difference in mean scores (95% CI)	P value	Difference in mean scores* (95% CI)	P value	Difference in mean scores† (95% CI)	P value
	on ≥3 consecutive days	78.80 (16)	76.76 (25)	-2.04 (-10.20 to 6.12)	0.616	-1.80 (-11.23 to 7.62)	0.701	0.06 (-10.46 to 10.58)	0.991
>14 mmol/L	at least once	81.38 (91)	77.86 (42)	-3.51 (-8.54 to 1.51)	0.169	-2.94 (-8.89 to 3.01)	0.330	-1.97 (-7.64 to 3.69)	0.492
	on ≥2 consecutive days	81.16 (29)	77.90 (27)	-3.26 (-10.35 to 3.83)	0.360	-3.22 (-11.45 to 5.00)	0.435	-3.60 (-11.43 to 4.24)	0.361
	on ≥3 consecutive days	70.00 (5)	77.94 (16)	7.94 (-4.64 to 20.52)	0.202	8.15 (-6.27 to 22.58)	0.251	16.90 (1.34 to 32.45)	0.035

Random intercepts were used to account for the clustering effects of twins/triplets and treating hospital.

* Adjusted for duration of hyperglycaemia (according to respective definition) during the first 28 postnatal days.

† Adjusted for gestational age at birth, durations of mechanical ventilation and steroid treatments during the first 14 postnatal days, surgery due to patent ductus arteriosus or necrotising enterocolitis during the first 14 postnatal days, and duration of hyperglycaemia (according to respective definition) during the first 28 postnatal days.
