# Supplementary material for: Neonatal hyperglycaemia is associated with worse neurodevelopmental outcomes in extremely preterm infants
Source: Arch Dis Child Fetal Neonatal Ed. 2021 Apr 16;106(5):460–6. doi: 10.1136/archdischild-2020-319926 (PMC8394751; doi:10.1136/archdischild-2020-319926)
Supplement: Supplementary data [file archdischild-2020-319926supp007.pdf]

Online supplementary table 7. Univariable and multivariable generalised linear mixed models for the associations between neonatal hyperglycaemia (>12 and >14 mmol/L) and Motor Assessment Battery for Children 2 (MABC-2) score at 6.5 years of age in children born extremely preterm.

**Hyperglycaemia definition**		**Mean MABC-2 score**		**Univariable**		**Multivariable**	
		**No hyperglycaemia (n)**	**Hyperglycaemia (n)**	**Difference in mean scores (95% CI)**	**P value**	**Difference in mean scores (95% CI)**	**P value**
**>12 mmol/L**	at least once	70.01 (174)	61.37 (171)	-8.64 (-12.45 to -4.82)	<0.001	-4.88 (-8.81 to -0.95)	0.015
	on ≥2 consecutive days	67.95 (256)	59.07 (89)	-8.88 (-13.28 to -4.47)	<0.001	-5.84 (-10.29 to -1.40)	0.010
	on ≥3 consecutive days	66.65 (303)	58.62 (42)	-8.03 (-13.86 to -2.19)	0.007	-2.05 (-7.86 to 3.76)	0.489
	Days with hyperglycaemia (per 1 day increase)	-	-	-1.37 (-1.92 to -0.83)	<0.001	-0.93 (-1.52 to -0.34)	0.002
**>14 mmol/L**	at least once	69.40 (219)	59.28 (126)	-10.12 (-14.08 to -6.16)	<0.001	-6.90 (-10.95 to -2.85)	0.001
	on ≥2 consecutive days	66.93 (291)	58.86 (54)	-8.07 (-13.26 to -2.87)	0.002	-3.10 (-8.26 to 2.05)	0.237
	on ≥3 consecutive days	66.62 (324)	50.93 (21)	-15.69 (-23.46 to -7.93)	<0.001	-10.73 (-18.38 to -3.09)	0.006

Hyperglycaemia definition	Mean MABC-2 score		Univariable		Multivariable	
	**No hyperglycaemia (n)**	**Hyperglycaemia (n)**	**Difference in mean scores (95% CI)**	**P value**	**Difference in mean scores (95% CI)**	**P value**
Days with hyperglycaemia (per 1 day increase)	-	-	-2.00 (-2.73 to -1.28)	<0.001	-1.47 (-2.21 to -0.72)	<0.001

A random intercept was used to account for the clustering effect of twins/triplets. Adjusted for gestational age at birth, sex, occurrence of intraventricular haemorrhage grade 3-4 and/or periventricular leukomalacia, durations of mechanical ventilation and steroid treatments during the first 14 postnatal days, surgery due to patent ductus arteriosus or necrotising enterocolitis during the first 14 postnatal days, number of culture-verified sepsis episodes during the first 14 postnatal days, educational status of the mother, and age at follow-up. MABC-2 total scores ≤75 and ≤68 are equivalent to ≤15th centile (borderline motor impairment) and ≤5th centile (developmental coordination disorder) in Swedish children, respectively.
