# Supplementary material for: Neonatal hyperglycaemia is associated with worse neurodevelopmental outcomes in extremely preterm infants
Source: Arch Dis Child Fetal Neonatal Ed. 2021 Apr 16;106(5):460–6. doi: 10.1136/archdischild-2020-319926 (PMC8394751; doi:10.1136/archdischild-2020-319926)
Supplement: Supplementary data [file archdischild-2020-319926supp008.pdf]

Online supplementary table 8. Univariable and multivariable generalised linear mixed models for the associations between insulin treatment in hyperglycaemic infants and Movement Assessment Battery for Children (MABC-2) score at 6.5 years of age in children born extremely preterm.

**Hyperglycaemia definition**		**Mean MABC-2 score**		**Univariable**		**Multivariable**			
		**No insulin (n)**	**Insulin (n)**	**Difference in mean scores (95% CI)**	**P value**	**Difference in mean scores* (95% CI)**	**P value**	**Difference in mean scores† (95% CI)**	**P value**
**>8 mmol/L**	at least once	66.21 (257)	54.44 (44)	-11.77 (-17.75 to -5.79)	<0.001	-5.66 (-12.79 to 1.47)	0.119	-3.75 (-10.92 to 3.42)	0.304
	on ≥2 consecutive days	64.76 (172)	55.07 (44)	-9.69 (-16.06 to -3.31)	0.003	-4.98 (-12.33 to 2.37)	0.183	-3.46 (-10.96 to 4.03)	0.363
	on ≥3 consecutive days	64.03 (101)	53.68 (40)	-10.36 (-17.32 to -3.39)	0.004	-7.85 (-16.18 to 0.48)	0.065	-5.49 (-14.66 to 3.67)	0.238
**>10 mmol/L**	at least once	65.08 (187)	54.54 (44)	-10.54 (-16.88 to -4.20)	0.001	-3.84 (-11.40 to 3.72)	0.318	-0.85 (-8.62 to 6.91)	0.829
	on ≥2 consecutive days	62.80 (99)	54.27 (43)	-8.53 (-15.31 to -1.74)	0.014	-3.96 (-11.70 to 3.79)	0.315	-1.62 (-9.95 to 6.72)	0.702
	on ≥3 consecutive days	62.27 (53)	53.26 (36)	-9.01 (-17.22 to -0.80)	0.032	-6.50 (-16.16 to 3.16)	0.185	-3.55 (-13.97 to 6.86)	0.499
**>12 mmol/L**	at least once	63.06 (128)	55.27 (43)	-7.79 (-14.61 to -0.96)	0.026	-3.41 (-11.77 to 4.96)	0.422	-0.83 (-9.36 to 7.70)	0.848
	on ≥2 consecutive days	62.59 (51)	55.31 (38)	-7.28 (-15.12 to 0.56)	0.068	-5.22 (-14.27 to 3.84)	0.255	-3.11 (-12.96 to 6.74)	0.532

Hyperglycaemia definition		Mean MABC-2 score		Univariable		Multivariable			
		No insulin (n)	Insulin (n)	Difference in mean scores (95% CI)	P value	Difference in mean scores* (95% CI)	P value	Difference in mean scores† (95% CI)	P value
	on ≥3 consecutive days	67.80 (17)	53.72 (25)	-14.08 (-26.66 to -1.50)	0.029	-10.54 (-25.03 to 3.95)	0.149	-7.42 (-22.75 to 7.91)	0.332
>14 mmol/L	at least once	60.50 (85)	55.17 (41)	-5.33 (-13.16 to 2.50)	0.180	1.47 (-6.94 to 9.87)	0.730	3.21 (-5.57 to 11.99)	0.470
	on ≥2 consecutive days	65.00 (27)	53.83 (27)	-11.17 (-20.83 to -1.51)	0.024	-4.91 (-15.68 to 5.86)	0.364	-4.21 (-14.51 to 6.09)	0.414
	on ≥3 consecutive days	63.80 (5)	48.94 (16)	-14.86 (-38.10 to 8.38)	0.197	-14.93 (-42.88 to 13.03)	0.276	-13.33 (-42.79 to 16.13)	0.346

Random intercepts were used to account for the clustering effects of twins/triplets and treating hospital.

* Adjusted for duration of hyperglycaemia (according to respective definition) during the first 28 postnatal days and age at follow-up.

† Adjusted for gestational age at birth, durations of mechanical ventilation and steroid treatments during the first 14 postnatal days, surgery due to patent ductus arteriosus or necrotising enterocolitis during the first 14 postnatal days, and duration of hyperglycaemia (according to respective definition) during the first 28 postnatal days.
